# Fluid balance dynamics and early postoperative outcomes in orthotopic liver transplantation: a prospective cohort study

**DOI:** 10.1016/j.bjane.2025.844619

**Published:** 2025-04-04

**Authors:** Suzana Margareth Lobo, Pedro Saggioro Paulucci, Lucas Martins Tavares, Graziela Benardin Luckemeyer, Luana Fernandes Machado, Neymar Elias de Oliveira, Silvia Prado Minhoto, Rita Cassia Alves Silva, Renato Ferreira da Silva, Marlon Souza Freitas, Francisco Ricardo Marques Lobo, Joana Berger-Estilita

**Affiliations:** a Hospital de Base da Faculdade de Medicina de São José do Rio Preto (FAMERP), Divisão de Terapia Intensiva, São José do Rio Preto, SP, Brazil; b Hospital de Base da Faculdade de Medicina de São José do Rio Preto (FAMERP), Divisão de Transplantes, São José do Rio Preto, SP, Brazil; c Institute of Anaesthesiology and Intensive Care, Salem Spital, Hirslanden Hospital Group, Switzerland; d Institute for Medical Education, University of Bern, Switzerland; e University of Porto, Faculty of Medicine, Centre for Health Technology and Services Research, CINTESIS@RISE, Porto, Portugal

**Keywords:** Fluid balance, Mortality, Organ dysfunction, Orthotopic liver transplant, Sequential organ failure assessment, SOFA

## Abstract

**Introduction:**

This study evaluates the impact of Fluid Balance (FB) patterns on outcomes after Orthotopic Liver Transplantation (OLT). It hypothesizes that deviations from optimal FB increase morbidity.

**Methods:**

In a single-center cohort post hoc analysis of 73 post-OLT patients, FB was categorized into three groups based on cumulative FB at 72 hours: Lowest (negative FB), Intermediate (0-2000 mL), and Highest (> 2000 mL). We analyzed Sequential Organ Failure Assessment (SOFA) scores, mortality rates, and causes of death. Logistic regression identified mortality predictors.

**Results:**

The Highest FB group had the highest SOFA scores and mortality (Group “Lo”: 18.2%, Group “In”: 8.6%, Group “Hi”: 40.5%, p = 0.009). A U-shaped relationship between FB and hospital mortality was observed, with extremes of FB associated with higher mortality. Cumulative FB independently predicted all-cause mortality with a 29.5% increase in the risk of death. FB on day 3 also predicted all-cause mortality, increasing the risk by 83.9%. Furthermore, FB on day 1 was linked to a 134.5% increase in the risk of death due to primary non-function of the liver. SOFA_LIVER_ score strongly predicted all-cause mortality, with a one-point increase associated with a 98.8% to 114.7% increase in mortality risk.

**Discussion:**

These findings suggest that both negative and positive extremes of FB are associated with worse outcomes after OLT, reinforcing the U-shaped relationship between FB and mortality. Our results underscore the importance of balanced fluid management, particularly in the early postoperative period. The study highlights the need for individualized FB strategies to optimize organ function and reduce mortality. The use of SOFA_LIVER_ scores as a predictor of mortality further emphasizes the importance of liver function monitoring in post-OLT patients. However, the single-centre design and convenience sample limit the generalizability of our findings, necessitating validation through multicenter studies.

**Conclusion:**

Our study provides valuable insights into the relationship between FB patterns and mortality in OLT patients. Both negative and positive extremes of FB are associated with higher mortality, suggesting the need for a balanced and individualized fluid management approach. The strong predictive value of SOFA_LIVER_ scores for all-cause mortality highlights the importance of early and continuous monitoring of liver function. Future multicenter randomized controlled trials are needed to validate these findings and develop optimized fluid management protocols for OLT patients.

## Introduction

Liver transplantation is a life-saving procedure for patients with end-stage liver disease, offering a chance for improved quality of life and survival.^1^ However, the perioperative period poses significant challenges, including managing Fluid Balance (FB), which can profoundly affect patient outcomes.^2^ Fluid overload, in particular, has emerged as a recognized risk factor in critically ill patients, contributing to organ dysfunction and mortality.^3^, ^4^, ^5^, ^6^ While the detrimental effects of fluid overload are well-documented in various clinical settings, such as sepsis,^7^, ^8^, ^9^ trauma,^10^^,^^11^ and gastrointestinal surgeries,^1^, ^2^, ^3^ its implications in the context of Orthotopic Liver Transplantation (OLT) remain less explored.^15^

Studies in major surgeries have shown that both hypovolemia and hypervolemia can lead to worse outcomes and more complications, potentially due to inadequate hemodynamic optimization during the perioperative period.^16^ Evidence suggests a U- or J-shaped relationship between risk and volume loading, where perioperative risk decreases with increasing volume load until a critical point, beyond which further volume loading escalates the risk of morbidity and mortality.^13^^,^^17^ However, few studies have examined the impact of volume loading in the early postoperative period of OLT in adults (OLT).^15^^,^^18^, ^19^, ^20^ Abrupt shifts in vascular tone, volume status, and inflammatory responses characterize this period, significantly affecting organ perfusion and recovery. It is particularly relevant to study this period because it coincides with the first phases of fluid resuscitation, which typically unfold within the first 48 to 72 hours: resuscitation (rapid fluid administration for stability), optimization (fine-tuning for perfusion) and stabilization (maintaining balance and preventing overload).^13^ Careful fluid management during this window may help avoid fluid overload, potentially facilitating a more efficient transition through these phases and improving overall recovery.

We postulate that similar patterns of worse outcomes associated with extremes of FB may occur after liver transplants. This study aims to evaluate FB patterns in the early postoperative period and their correlation with organ dysfunction/failure and mortality following liver transplantation. We hypothesize that deviations from optimal FB may contribute to adverse outcomes, including organ dysfunction and mortality. Furthermore, we aim to identify potential predictors of mortality, including cumulative FB and its temporal evolution in the early post-transplant period.

## Material and methods

### Study design

This is an exploratory post hoc analysis of an observational cohort study^4^ in a convenience sample of adult patients consecutively admitted to an ICU from a university public tertiary hospital.

### Ethics

The study was approved by the institutional review board and the local institutional ethics committee (approval number CAAE 51448015.8.0000.5415). Before inclusion, written informed consent was obtained from the patient or the next of kin. In reporting, we followed the STROBE guidelines.^21^

### Participants

All consecutive adult patients (> 18 years) admitted postoperatively after OLT in the ICU of São José do Rio Preto, São Paulo, Brazil, from December 1^st^, 2015, to December 31^st^, 2016, were considered for inclusion in the study. Exclusion criteria included acute or chronic kidney disease, hepatorenal syndrome, reoperation, and ICU LOS lower than 48h.

### Outcomes

The primary outcome was cumulative FB in the first three days after Orthotopic Liver Transplantation (OLT) and its relation to organ dysfunction/failure and hospital all-cause mortality. Secondary endpoints included evaluating Sequential Organ Failure Assessment (SOFA) scores as a measure of organ dysfunction concerning accumulated FB and comparing SOFA scores between survivors and non-survivors to assess the impact of FB on organ function and mortality.

### Data collection

The primary study^20^ describes data collection and management, and we prospectively collected all data, ensuring no missing data. In brief, we gathered various parameters related to FB, organ function, and clinical outcomes in patients admitted to the ICU after OLT. This included age, comorbidities, type of surgery, American Society of Anesthesiologists Physical Status (ASA-PS) classification,^22^ MELD Score,^23^ Simplified Acute Physiology Score III scores (SAPS3)^24^ on admission, daily use of vasoactive drugs and mechanical ventilation. We also calculated the cumulative FB over 3 days. Parameters such as serum creatinine levels, urine output, and SOFA scores^25^ (assessing Cardiovascular [CV], Respiratory [RESP], coagulation, liver, neurologic, and renal systems) were also monitored daily. Based on predefined criteria, we defined Intra-Abdominal Hypertension (IAH), Abdominal Compartment Syndrome (ACS), Acute Kidney Injury (AKI), and organ failure. We recorded admission data from the day of ICU admission until 23:59 hours as Day 1 (D1), Day 2 (D2), and Day 3 (D3) referred to the subsequent two days.

Daily FB was the daily sum of all intakes (such as crystalloids, colloids, drugs in solution, blood derivatives, and fluids via nasogastric tube) minus the output (diuresis, bleeding, dialysis, and drainage). The cumulative FB was the sum of daily FB over 3 days.^20^^,^^26^ Furthermore, we assessed intra-abdominal pressure values concerning different FB groups. Patients were stratified into three distinct groups according to their patterns of accumulated FB after 72 hours of liver transplantation: Group Lowest (“Lo”) ‒ negative FB, Group Intermediate (“In”) ‒ FB: 0 –2000 mL; Group Highest (“Hi”) ‒ FB > 2000 mL.

SOFA scores were determined on D1, D2 and D3. Hi and mean SOFA scores were calculated using the 3-day FB values.^27^ Delta SOFA D2 was SOFA D2 minus SOFA D1. Delta SOFA D3 was SOFA D3 minus SOFA D1. We followed patients until discharge or death, monitoring parameters and outcomes throughout their hospital stay.

The causes of mortality, such as sepsis, Multiple Organ Failure (MOF), and Primary Non-Function (PNF) of the liver, were retrieved from the case record forms.^20^

### Statistics

We presented categorical variables as frequencies and percentages and quantitative variables as medians and Interquartile Ranges (IQR). We analyzed categorical variables using Pearson's Chi-squared test (χ^2^) or Fisher's exact test. We analyzed continuous variables using the Kruskal-Wallis test adjusted for ties or using General Linear Model (GLM) with random effects for data with a non-Gaussian distribution. Univariate and multivariate logistic regression (forward stepwise) analyses were performed to determine the independent predictors of all-cause mortality and mortality due to PNF of the liver (dependent variables). The independent variables selected for entering the binary logistic regression analysis were chosen by a p-value < 0.25 in the univariate analysis for all-cause mortality and death to PNF (Table S1). The variables tested were age, SOFA_LIVER_, SOFA_RESP_, FB D1, FB d3 and accumulated FB. MELD was forced in the model despite p > 0.25. We evaluated the variables separately, first for accumulated FB (Model 1) and then for FB on D1 and D3 (Model 2) as independent predictors of death in the logistic regression to determine when fluid use became more deleterious. We conducted Variance Inflation Factor (VIF) analysis to test multicollinearity for all covariates. VIF < 1.5 suggests no multicollinearity. While for multivariate analysis, a ratio of 1 covariate per 10 to 20 outcomes is usually preferred, we followed the recommendations from van Smeden et al.,^28^ which indicates no rigid justification for the ratio of 1 covariate per 10 events for binary logistic regression analysis. The adjusted Odds Ratio (OR) and 95% Confidence Intervals (95% CIs) were calculated for the predictors. We considered a p-value < 0.05 statistically significant. The software packages used for statistical analyses included IBM SPSS Statistics (version 25.0), *R* (version 3.4.1) and Minitab 17 Statistical Software.

## Results

**Figure 1:**
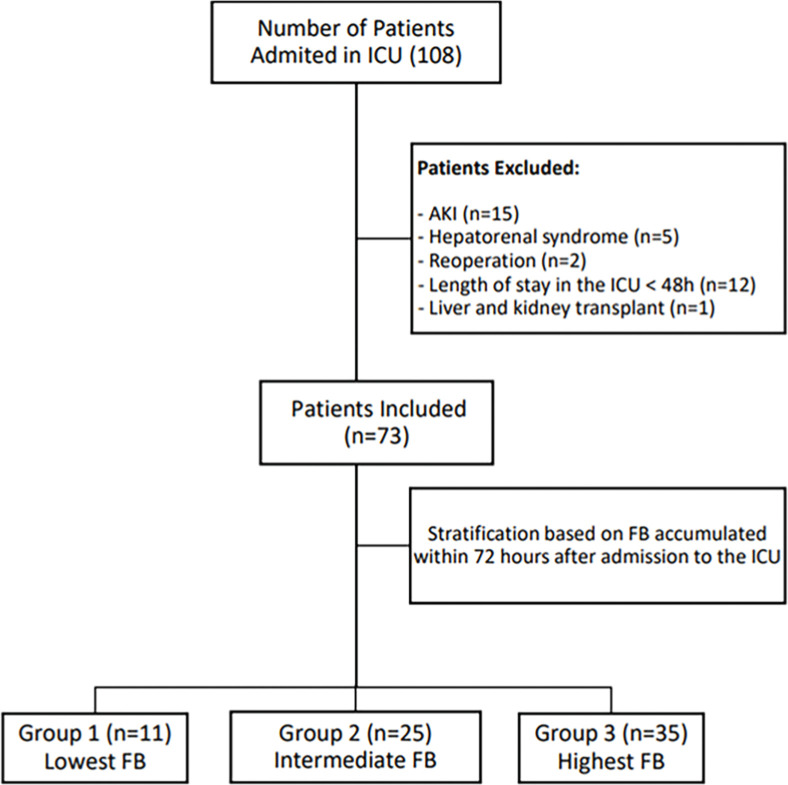
Flowchart, depicting the selection and stratification process of the study's patients. AKI, Acute Kidney Injury; FB, Fluid Balance; ICU, Intensive Care Unit.

During the study period, we admitted 108 patients to the ICU after OLT and enrolled 73 of them. Twenty-five patients were excluded, including those with acute or chronic kidney disease (n = 15), length-of-stay lower than 48h (n = 12), hepatorenal syndrome (n = 12), reoperation (n = 2), and liver and kidney transplant (n = 1) (Fig. 1).

Among the 73 patients included in the study, the mean age was 51.5 ± 12 years and the median [IQR] MELD 17^7^, ^8^, ^9^, ^10^, ^11^, ^12^, ^13^, ^14^, ^15^, ^16^, ^17^, ^18^, ^19^, ^20^, ^21^, ^22^, ^23^, ^24^, ^25^, ^26^, ^27^, ^28^, ^29^, ^30^, ^31^, ^32^, ^33^, ^34^, ^35^, ^36^, ^37^ (Table 1). A total of 11 patients (15%) were included in Group “Lo” with a median [IQR] accumulated FB of -184 mL [-992, -144 mL], 25 patients (34.2%) in Group “In” with 1167 mL [784,1167 mL] and 35 patients (50.7%) in Group “Hi” with 3726 mL [3144‒5729 mL]. Groups “In” and “Hi” presented greater intra-abdominal pressure values with a trend toward statistical significance in comparison to the “Lo” Group.

**Table 1:** Baseline clinical characteristics, SOFA scores and mortality in the groups according to accumulated fluid balance.

	All (n = 73)	Group Lowest “Li” (n = 11)	Group Intermediate “In” (n = 25)	Group Highest “Hi” (n = 37)	p-value
**Age, years**		53 (48-61)	57 (48-60)	52 (43-58)	0.341
**Weight, Kg**		75.6 ± 17.3	70.9 ± 17.3	79.1 ± 17.4	0.847
**FB cumulative (L)**		-0.2 [-1.0‒0.1]	1.2 [0.8‒1.4]	3.7 [3.1‒5.7]	**< 0.001**
**MELD**		16 [10-21]	15.9 [12-20]	17.4 [11-21]	0.47
**Child A (n, %)**		3 (27%)	9 (36%)	11 (29%)	**0.046**
**Child B (n, %)**		3 (27%)	4 (16%)	16 (43%)	0.385
**Child C (n, %)**		5 (45%)	12 (48%)	10 (27%)	0.929
**IAP D1**		9.0 ± 3.4	11.0 ± 3.9	12.3 ± 4.1	0.054
**IAP D2**		10 ± 1.6	11 ± 3.7	12.6 ± 4.1	0.073
**IAP D3**		11 ± 6.9	11.6 ± 4.1	12.6 ± 4.4	0.489
**SOFA scores and outcomes**					
SOFA D1	8 [6; 11]	6 [5,8]	8 [6,10]	10 [5,12]	0.113
SOFA D2	7 [5,11]	6 [4,10]	6 [4.5, 9]	9 [5,12]	0.061
SOFA D3	7 [5,10]	6 [5,8]	5 [5,7.5]	7 [6,11]	**0.021**
SOFA mean	7.3 [5.7, 10.0]	6.0 [4.7, 9.3]	6.3 [5.7, 8.7]	8.3 [5.8, 12.0]	**< 0.001**
SOFA max*	8 [5.5, 9.0]	6 [6,10]	8 [6,10]	10 [7,13]	**0.013**
Delta SOFA D2	0 [-2, 1]	0 [0; 2]	-1 [-2;1]	0 [-2, 1]	0.193
Delta SOFA D3	0 [-3; 1.5]	0 [-2; 1]	-1 [-3; 0.5]	0 [-3; 2]	0.281
SOFA _CV_ D1	3 [0,4]	0 [0,3]	4 [0,4]	3 [2,4]	**0.026**
SOFA _CV_ D2	0 [0,3]	0 [0,0]	0 [0,3]	3 [0,3, 5]	0.067
SOFA _CV_ D3	0 [0; 1, 5]	0 [0,0]	0 [0,0]	0 [0,3]	0.060
SOFA _RESP_ D1	1 [0,1]	1 [0,1]	1 [0,1]	1 [0,2]	0.126
SOFA _RESP_ D2	1 [0,1]	0 [0,1]	0 [0,1]	1 [0,1]	0.585
SOFA _RESP_ D3	1 [0,1]	0 [0,1]	0 [0,1]	1 [0,1]	0.348
SOFA _RENAL_ D1	1 [0,1]	0 [0,1]	1 [0,1]	1 [0,2]	0.446
SOFA _RENAL_ D2	1 [0,2]	1 [0,2]	1 [0,2]	1 [0,5; 2]	0.390
SOFA _RENAL_ D3	1 [0,2]	1 [0,2]	1 [0,2]	1 [1,2]	0.208
**LOS ICU, (Days)**	3 [2,6]	3 [2,6]	2 [1,5]	5 [2,6]	0.129
**Mortality rate (%)**	19 (26.0)	2 (18.2)	2 (8.6]	15 (40.5)	**0.009**
**Death due to PNF (%)**	8 (11.0)	1 (9.1)	0 (0)	7 (18.9)	**0.023**

MELD, Model for End-Stage Liver Disease; IAP, Intra-Abdominal Pressure; SOFA, Sequential Organ Failure Assessment; FB, Fluid Balance. Liter, L; LOS, Length of Stay; PNF, Primary Non-Function of the liver. Numbers are presented as n (%) or median and 25%; 75% IQR.

### SOFA scores according to accumulated fluid balance

**Figure 2:**
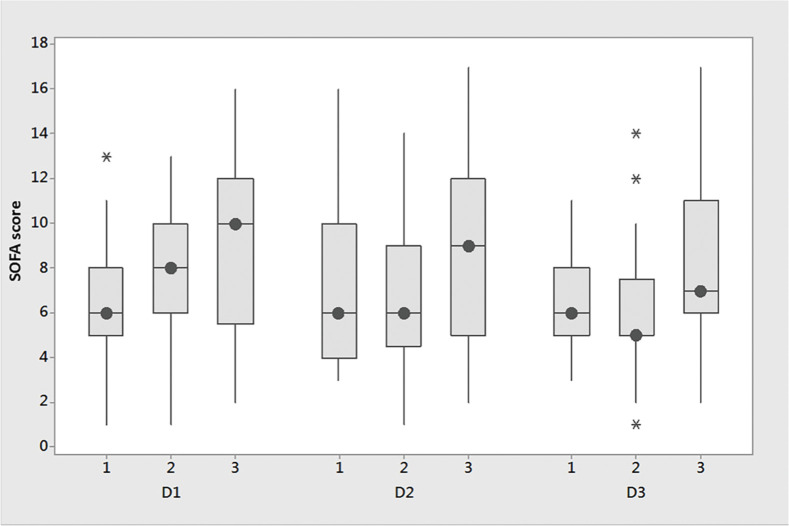
Boxplot of SOFAD1_1; SOFAD2_1; SOFAD3 in the groups ‘Lowest”, ‘Intermediate’ and ‘Highest’ FB. The image is a box plot that compares SOFA scores across three different time points: D1, D2, and D3. The box plots for each group show the median SOFA score (the line inside the box), the Interquartile Range (IQR, represented by the length of the box), and the range (the “whiskers” extending from the top and bottom of the box). Asterisks indicate outliers. The small circles within the boxes represent the mean SOFA scores. The SOFA score is plotted on the y-axis, ranging from 0 to 18. For each time point (D1, D2, D3), the plot labeled ‘1’ correspond to group Lowest, ‘2' to Group Intermediate, ‘3’ group Highest. D1: Day 1; D2: Day 2; D3: Day 3; SOFA, Sequential Organ Failure Assessment.

Median [IQR] SOFA scores for the 3 groups on days 1, 2, and 3 are shown in Table 1 and Figure 2. Group “Hi” had higher SOFA score values on days 1, 2 and 3. Group “In” had decreasing SOFA scores on days 2 and 3 relative to Day 1 and negative values for delta SOFA D2 and delta SOFA D3. Group “Hi” had the highest value of mean SOFA score (Group “Lo”: 6 [4.7, 9.3], Group “In”: 6.3 [5.7, 8.7], Group “Hi”: 8.3 [5.8, 12.0]; p < 0.001). Group “Hi” had a significantly higher SOFA_max_ on day 3 (7 [6‒11]) in comparison to groups “Lo” (6 [6,10]) and “In” (5 [5‒7.5]) (p = 0.021). On day 1, the cardiovascular SOFA score was significantly higher in groups “In” (4 [0‒4] and “Hi” (3 [2‒4] than in Group “Lo” (0 [0‒3]) (p = 0.026) but declined from a median of 4 to 0 in Group “In”, but not in Group “Hi” (Table 1). There were no significant differences between the respiratory and renal components of the SOFA score.

### FB and SOFA scores in survivors and non-survivors

**Figure 3:**
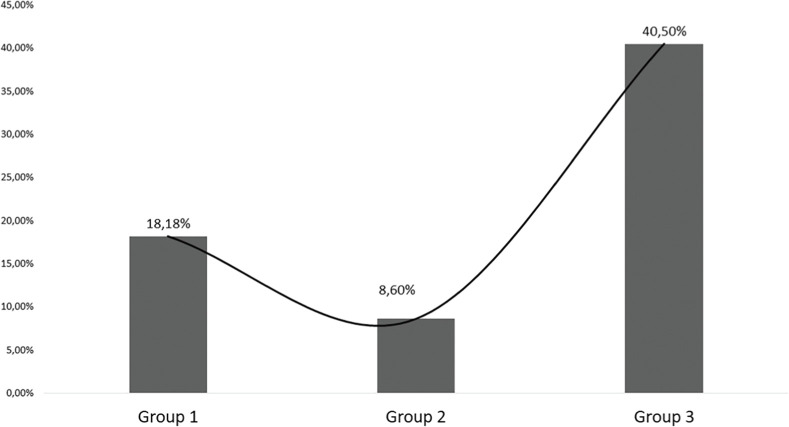
Hospital mortality rates of OLT patients after ICU admission. The image is a bar chart with an overlaid line graph that illustrates the mortality rates for three different groups of patients. Each bar represents a group, labeled as Group 1 (“Lo”), Group 2 (“In”), and Group 3 (“Hi”). The mortality rates are indicated by the height of the bars as well as by percentages labelled on the corresponding points on the line graph, which connects these percentages across the groups. The y-axis of the chart indicates the mortality rate percentages, ranging from 0% to 45%.

**Figure 4:**
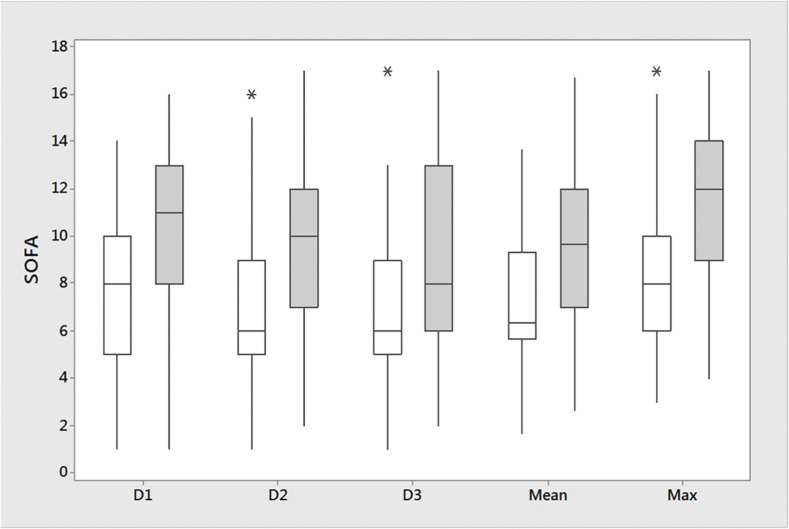
Box plot of SOFA scores in survivors and non-survivors. Image of a box plot comparing SOFA scores over time between survivors (White) and non-survivors (Gray). This plot includes several boxes that represent different time points (D1, D2, D3), along with an aggregate measure of the mean and the maximum values. Each box plot shows the median SOFA score (indicated by the line inside the box), the interquartile range (the box itself), and the overall range excluding outliers (the “whiskers” extending from the top and bottom of the box). Outliers are marked with asterisks. The circles inside represent the median [25%‒75%] SOFA scores for each category. D1: Day 1; D2: Day 2; D3: Day 3; SOFA, Sequential Organ Failure Assessment.

A total of 19 patients (26%) died (Group “Lo”: two patients (18.2%), Group “In”: two patients (8.6%), Group “Hi”: 15 patients (40.5%), p = 0.009) (Table 1, Fig. 3). The leading cause of death was sepsis and MOF in 11 patients (one patient in Group “Lo”, two patients in Group “In” and eight patients in Group “Hi”). Primary non-function was the cause of death in 8 patients (9%), [one patient in Group “Lo” and seven patients in Group “Hi” (19%), p = 0.023, Fig. 4]. Table S1 show the clinical characteristics, FB, and SOFA score in patients who were discharged alive versus those who experienced all-cause mortality or death due to PNF of the liver. Patients who died had a significantly higher accumulated three days FB with 3542 mL [2018‒5847 mL] vs. 1467 mL [513‒3409 mL] (p = 0.013). Total SOFA scores were significantly higher in non-survivors than in survivors (day 1: 11 [8‒13] vs. 8 [5-8], p = 0.009, day 2: 12 [7-12] vs. 6 [5‒9], p = 0.006, day 3: 8 [6‒13] vs. 6 [5‒9], p = 0.013, survivors vs. non-survivors, respectively). Mean SOFA score (9.7 [7‒12] vs. 6.1 [5.7‒13.7], p = 0.005) and SOFA_max_ score (12 [9-14] vs. 8 [6-10], p = 0.003) were also significantly higher in non-survivors (Table 1).

### Independent predictors of mortality

To determine predictive relationships, binary logistic regression analysis was performed. Cumulative FB was an independent predictor of all-cause mortality (OR = 1.295, 95% CI 1.024; 1.638, p = 0.026). FB-D3 predicted all-cause mortality (OR = 1.839, 95% CI 1.0003‒3.509; p = 0.046) (Table 2). FB-D1 was predictive of death due to primary-non function of the liver (OR = 2.345, 95% CI 1.132; 4.860, p = 0.014). SOFA_LIVER_ predicted all-cause mortality (Model 1: OR = 1.988, 95% CI 1.181; 3.345; p = 0.006; Model 2: OR = 2.147, 95% CI 1.239; 3.7215, p = 0.003).

**Table 2:** Logistic regression analysis with all-cause mortality and mortality due to primary non-function of the liver as dependent variables.

	Univariate			Multivariate		
Variables	OR	95% CI	p-value	OR	95% CI	p-value
**All-cause mortality**						
**Model 1**						
Cum FB	1.289	1.005; 1.654	**0.039**	1.295	1.0248; 1.6381	**0.026**
SOFA_RESP_	1.041	0.492; 2.149	0.916			
SOFA_HEPA_	2.054	1.171; 3; 603	0.007	1.988	1.181; 3.345	**0.006**
MELD	0.979	0.891; 1.077	0.676			
**Model 2**						
FB D1	1.353	0.789; 2.319	0.275			
FB D3	1.919	0.979; 3.762	**0.039**	1.839	1.0003; 3.509	**0.046**
SOFA_RESP_	1.00594	0.4593; 2.4440	0.892			
SOFA_HEPA_	2.2510	1.2305; 4.1178	0.039	2.1479	1.2397; 3.7215	0.003
MELD	0.9746	0.8784; 1.0814	0.628			
**Primary non-function**						
Model 2						
FB D1	1.853	0.778; 4.409	0.168	2.345	1.132; 4.860	**0.014**
FB D3	2.655	0.858; 7.665	**0.059**			
Age	0.9312	0.8634; 1.0043	0.059			
SOFA_RESP_	1.9162	0.5256; 6.9861	0.352			
MELD	0.9336	0.7960; 1.0950	0.398			

All VIF values were close to 1, indicating that multicollinearity is not a concern in the regression model, and there is no strong linear association among the independent variables. Model 1 included Cumulative Fluid balance (Cum FB) and Model 2 daily Fluid Balance (FB D1 and FB D3). SOFA total, mean or max were not entered in the model due to multicollinearity (high VIF).

### Post-hoc power analysis

As our study was based on a convenience sample, a post-hoc power analysis was conducted to evaluate its ability to detect observed mortality differences between groups. The power is low (19%–32%) for detecting differences between Group 1 and Group 2, indicating a high risk of a Type II error. It increased to moderate (55%–69%) for comparisons between Group 1 and Group 3, but remained insufficient for strong conclusions. In contrast, the power was high (97%–99%) for Group 2 vs. Group 3, suggesting a reliable detection of differences.

## Discussion

This exploratory study's main findings indicate that lower and higher extremes of FB were associated with a greater degree of organ failure over time and increased mortality rates. Cumulative FB emerged as an independent predictor of all-cause mortality, as well as mortality specifically due to PNF of the liver. Additionally, FB on day 3 was independently associated with all-cause mortality. In contrast, FB on days 1 and 3 was independently linked to primary liver dysfunction or failure as a cause of death.

Higher values of SOFA score mean SOFA and SOFA_max_ in patients from Group “Hi” clearly indicated an association between a higher degree of organ dysfunction/failure over time and FB. The mean SOFA score indicates the average degree of organ failure over time. SOFA_max_ is related to a critical point at which patients exhibit more organ dysfunction during their ICU stay. Calculations of delta SOFA D2 and delta SOFA D3 can point to improvements in organ function.^27^ Therefore, the negative values for delta SOFA in Group “In” on days 2 and 3 may indicate a quicker resolution of organ dysfunction/failure in this group and better outcomes. In addition, SOFA_CV_ declined from a median of 4 to 0 in Group “In” but not in Group “Hi”, which points to the fastest resolution of shock. Of relevance, the SOFA score is helpful in predicting adverse outcomes in high-risk liver recipients.^29^, ^30^, ^31^ Our study underscores the importance of closely monitoring and managing the liver function in the early post-transplant period SOFA_LIVER_ score was a strong predictor of all-cause mortality with one-point increase in SOFA_LIVER_ associated with a 98.8% to 114.7% increase in mortality risk. Accordingly, Hao-Chien Hung et al. investigated the predictive value of the SOFA score among high-risk patients post-living donor liver transplantation.^32^ Multivariate analysis identified elevated liver (HR = 10.4) and cardiovascular components (13.3) of SOFA score as predictors of death. By identifying patients with elevated SOFA scores, especially in these components, clinicians can implement targeted interventions to potentially improve outcomes after LDLT.

Our results suggest the presence of different phenotypes for FB associated to various outcomes, which could be identified early after ICU admission. A significantly higher proportion of patients died in the two extremes of FB. Tacker et al. reported similar results in a large cohort of GI and orthopedic surgeries with increased morbidity and costs for the highest and the lowest 25 percentile of the fluid volume received.^33^ They showed significant associations between high fluid volume on the day of surgery and increased length of stay and total costs. Stepwise increases in the hazard of complications or death with higher cumulative FB were reported in different populations of critically ill patients.^3^^,^^4^^,^^9^^,^^11^^,^^34^ In a prospective cohort study, Bennet-Guerrero et al. reported age and total intraoperative fluids as predictors of adverse outcomes in patients undergoing OLT.^35^ In their study, for each 1 L of fluids, the risk of adverse outcomes increased by 7%. In our study, excessive postoperative fluid administration increased the risk of PNF. Accordingly, Jiang et al. investigated the impact of individualized peri-operative fluid therapy on early-phase recovery following OLT in a retrospective analysis of 102 patients.^36^ They reported that achieving a negative FB of at least -14 mL.kg^−1^ on the first and second or third post-operative days was linked to fewer pulmonary complications, earlier extubation, and a quicker return of bowel function.

Patients who died had a significantly higher accumulated three days FB. In the logistic regression analysis, a more positive accumulated FB on D3 was an independent predictor of deaths. Excess fluids can generate a cycle of hepatic congestion, impaired microcirculation, intra-abdominal hypertension, and organ dysfunctions that, in turn, can affect their elimination and create a worsening of hypervolemia and MOF.^13^^,^^26^ Indeed, the SOFA_LIVER_ score strongly predicted all-cause mortality, with a one-point increase in SOFA_LIVER_ associated with a 98.8% to 114.7% increase in mortality risk. In a study by Larivière et al. involving 562 liver transplant patients, 3.2% developed PNF.^37^ A higher intraoperative FB was non-linearly associated with the harmful effect of a higher FB on primary graft non-function and survival. We observed a 29.5% increase in the risk of all-cause mortality for each 1 L of accumulated FB, an 84% increase in risk associated with FB on day 3, and more than twofold.

Our findings indicate that very early positive FB after ICU admission (days 1 and 3) is associated with death. In contrast, higher fluid use later on, appears more related to death from sepsis and MOF. Adopting a more restrictive fluid approach can lower postoperative complications in colorectal surgery patients,^38^^,^^39^ but this strategy isn't universally agreed upon in all surgeries. Froghi et al.^40^ demonstrated in a small randomized trial of fluid therapy post-liver transplant Goal-Directed Fluid Therapy (GDFT) was associated with an increased volume of crystalloids administered but did not alter early post-operative pulmonary or renal function when compared with standard care. This underscores the importance of focusing on blood flow stability to improve outcomes after surgery.

An important consideration in this study is the rationale behind the arbitrary definition of the three groups. While no universal consensus exists on the optimal FB thresholds, previous studies have underscored both the detrimental effects of fluid overload and the risks associated with excessive restriction. The negative FB group represents patients with a more restrictive fluid strategy. The intermediate group (0-2 L) was chosen as a plausible “neutral” range, avoiding significant depletion and excessive accumulation. The highest FB group (> 2 L) encompasses patients with notable fluid accumulation, a threshold frequently associated with adverse postoperative outcomes. Given our sample size, employing more granular stratifications, such as terciles or quartiles, was not statistically feasible. Instead, we selected cutoff points that provided meaningful distinctions and aligned with clinical practice and real-world decision-making. Another consideration is the rationale behind using actual weight instead of weight-adjusted measures for FB. In cirrhotic patients with ascites/edema, weight-adjusted measures such as ideal or dry weight are preferable to avoid overestimating FB. Using BMI for FB calculations is unreliable in end-stage liver disease, where ascites, peripheral edema, and malnutrition distort actual body composition, making BMI-based adjustments inaccurate and potentially biased results.^41^ If weight-adjusted values are unavailable, absolute cumulative FB may be a more practical metric, with acknowledgement of its limitations.

Effective postoperative fluid management in the ICU is crucial for recovery, with protocols varying by institution and patient needs. In the ICU, GDFT optimizes perfusion by tailoring fluids based on hemodynamic parameters. Ideally, fluid management should follow the four phases ‒ resuscitation, optimization, and stabilization ‒ to prevent fluid overload after 48h and minimize the need for the fourth phase, de-escalation, which involves removing excess fluid.^13^ This structured approach ensures systematic and individualized fluid therapy in critically ill patients and may help prevent complications, warranting further investigation in RCT. Based on our findings and other studies, we recommend this approach for OLT to optimize postoperative outcomes. Continuous monitoring of hemodynamic parameters, urine output, and laboratory values is essential, with fluid therapy individualized according to the patient's evolving clinical status, underlying conditions, and treatment response.^42^^,^^43^

Several limitations must be acknowledged. First, our study was based on a convenience sample from a single center, limiting the generalizability of our findings. This may introduce selection bias and restrict the applicability of our conclusions to other transplant centers with different perioperative practices. Additionally, the small sample size reduces the statistical power of subgroup analyses and increases the risk of Type II errors, potentially underestimating the effect sizes. Our FB thresholds were arbitrarily chosen, as finer stratifications were not feasible due to the limited sample size. Using absolute cumulative FB rather than weight-adjusted measures may not account for body composition differences, particularly in cirrhotic patients with ascites or edema. This could lead to potential misclassification of FB categories. Furthermore, the observational study design precludes definitive conclusions about causal relationships between FB patterns and clinical outcomes. We did not include intraoperative variables, such as blood loss and hemodynamic instability, which may have influenced postoperative FB and outcomes. Moreover, the study primarily focused on short-term outcomes, leaving long-term consequences unexplored. These limitations highlight the need for caution when interpreting the results and underscore the necessity for further multicenter Randomized Controlled Trials (RCTs) to validate our findings in more diverse populations.

Our study possesses several strengths. A major strength is the prospective data collection, which ensured the completeness and accuracy of clinical parameters. The study design allowed for a detailed analysis of FB patterns and their temporal evolution in the critical early postoperative period, providing valuable insights into the dynamic nature of FB and its impact on organ dysfunction and mortality. Our work addresses a significant gap in the literature by exploring the effects of FB on outcomes in OLT patients. This population remains understudied in the context of postoperative fluid management. By categorizing FB patterns and assessing their association with Sequential Organ Failure Assessment (SOFA) scores, we provide a more nuanced understanding of how FB extremes influence organ dysfunction and mortality. Additionally, our study is the first to identify SOFA_LIVER_ scores as a strong predictor of all-cause mortality, emphasizing the importance of liver function monitoring in the early postoperative phase.

Future research should focus on validating these findings through multicenter RCTs that incorporate intraoperative variables and long-term outcomes. Investigating the impact of weight-adjusted FB measures and exploring the role of dynamic hemodynamic monitoring in guiding FB strategies are also essential. Further studies should examine the underlying mechanisms driving the U-shaped relationship and evaluate the potential of personalized fluid management protocols tailored to individual patient characteristics. Our results provide a foundation for hypothesis generation and underscore the necessity for more robust clinical evidence to guide FB management in OLT patients. Ultimately, by optimizing fluid management strategies, we aim to improve postoperative outcomes and reduce mortality in this high-risk population.

## Conclusions

This study suggests that negative FB and fluid overload are associated with worse outcomes after OLT. Optimal fluid management in the early postoperative period is one of the most essential tasks of those caring for postoperative patients. The anesthesiologist and the intensivist should adequately manage the different phases of fluid therapy, starting with individualized strategies in the intraoperative period in patients and surgeries at higher risk and followed quickly by the other phases in the ICU. Multicenter collaborations are essential to validate our findings in broader and more diverse patient populations, including long-term follow-ups. Our findings should be considered hypothesis-generating rather than definitive, but they provide valuable insights into a population that remains understudied in the context of postoperative fluid management.

## Ethics

The study was approved by the institutional review board and the local institutional ethics committee (approval number CAAE 51448015.8.0000.5415).

## Authors' contributions

Conceptualization: RFS, SML. Data curation: RFS, SML. Formal analysis: MSF, FEN, MLM, RCS, FRL, NEO, RFS, SML. Investigation: RFS, SML, JBE. Methodology: MSF, FEN, MLM, RCS, FRL, NEO, RFS, SML, FRML. Project administration: RFS, SML. Resources: RFS, SML. Supervision: RFS, SML. Validation: JBE. Visualization: MSF, FEN, MLM, RCS, FRL, NEO, RFS, SML. Writing-original draft: RFS, SML, FRML. Writing-review & editing: RFS, SML, JBE, MSF, FEN, MLM, RCS, FRL, NEO, FRML.

## Funding

None.

## Declaration of competing interest

JBE is a member of the Save the Brain Initiative Steering Committee, supported by Medtronic®, and participates in the Speakers Bureau of Medtronic®. Additionally, she is Chair of the Education and Training Committee and a member of the Scientific Committee. The remaining authors declare to have no competing interests.

## Appendix Supplementary materials

Image, application 1

## Footnotes

Supplementary material associated with this article can be found in the online version at doi:10.1016/j.bjane.2025.844619.
