# Supplementary material for: Fluid balance dynamics and early postoperative outcomes in orthotopic liver transplantation: a prospective cohort study
Source: Braz J Anesthesiol. 2025 Apr 4;75(3):844619. doi: 10.1016/j.bjane.2025.844619 (PMC12047465; doi:10.1016/j.bjane.2025.844619)
Supplement: Supplementary file 1 [file mmc1.docx]

**BJAN-D-24-00591_ Supplementary Material**

**Supplementary Material** **Table S1** Clinical characteristics, fluid balance, and SOFA score in patients who were discharged alive versus those who experienced all-cause mortality or death due to primary non-function of the liver.

| **Variables** | **Alive (n = 54)** | **Deceased All-cause mortality (n = 19)** | **Alive (n = 62)^a^** | **Deceased Primary non-function (n = 8)** | **p-value^b^** | **p-value^c^** |
| --- | --- | --- | --- | --- | --- | --- |
| Age (years) | 52 ± 11 | 43 ± 16 | 52 ± 10 | 43 ± 17 | 0.538 | 0.192 |
| Weight (kg) | 76 ± 18 | 75±17 | 77 ± 18 | 71 ± 13 | 0.980 | 0.494 |
| MELD | 17 ± 6 | 17±9 | 17 ± 6 | 16 ± 9 | 0.816 | 0.410 |
| IAP (mm.Hg) | 11 [8; 13.2] | 11 [9; 16] | 11 [8; 13] | 11 [9; 16] | 0.322 | 0.496 |
| FB D1 (L) | 1.5 [0.5; 1.4] | 1.70 [1.2; 3.7] | 1.4 [0.5; 2.1] | 3.1 [1.6; 4.0] | 0.074 | 0.010 |
| FB D2 (L) | -0.1 [0.6; 1.4] | 0.5 [;0.5; 1.4] | 0.6 [0.1; 1.4] | 0.5 [-0.5; 1.4] | 0.831 | 0.941 |
| FB D3 (L) | 0.2 [-0.3; 0.6] | 1.8 [1.0; 2.2] | 0.2 [-0.4; 0.6] | 1.8 [1.0; 2.3] | 0.007 | 0.002 |
| Cumulative FB (L) | 1.9 [0.7;3.0] | 5.0 [2.5; 7.0] | 1.8 [0.7; 3.4] | 5.1 [2.5; 7.0] | 0.013 | 0.015 |
| SOFA_RESP_ | 1.0 [0.0; 1.0] | 1.0 [1.0; 2.0] | 1.0 [0.0; 1.0] | 1.0 [1.0; 2.0] | 0.208 | 0.074 |
| SOFA COAG | 2.0[2.0; 3.0] | 2.5 [1.2; 3.0] | 2[2; 3] | 2.5 [1.2; 3] | 0.935 | 0.647 |
| SOFA_CV_ | 3.0 [0.0; 4.0] | 3.0 [3.0; 4.0] | 3 [0; 4] | 3 [3; 4] | 0.370 | 0.544 |
| SOFA_LIVER_ | 2 [0.7; 2] | 1.5 [0.2; 3] | 2 [0.7; 2] | 1.5 [0.2; 3] | 0.009 | 0.703 |
| SOFA_SNC_ | 0 [0; 1] | 0 [0; 2] | 0 [0; 1] | 0 [0; 2] | 0.336 | 0.395 |
| SOFA_Renal_ | 1 [0; 1] | 1 [0; 2.75] | 1 [0; 2.7] | 1 [0; 3] | 0.463 | 0.288 |
| SOFA_Total_ D1 | 8 [5.7; 10] | 11 [6.5; 14] | 8 [6; 10] | 11 [6.5;11] | 0.009 | 0.055 |
| SOFAMean | 6 [5.7; 9] | 9.7 [7; 12] | 7 [5.7; 10] | 9.5 [7; 1 5] | 0.005 | 0.104 |
| SOFA_Max_ | 8 [6; 10] | 12 [8; 16] | 9 [7; 11] | 12 [9; 14] | 0.004 | 0.081 |
| SOFADelta 48h | 0 [-2; 1] | 0 [-2.0; 1.0] | 0 [-2 – 1] | 0 [-2 – 1] | 0.415 | 0.779 |
| SOFADelta 72h | 0 [-3; 1] | -1 [-3; 2] | 0 [-3 – 1] | 0.5 [-4.5 2] | 0.825 | 0.860 |

^a^ Three cases with uncertain causes of death were excluded from the group analysis of deceased non function of the liver.

^b^p-value: Deceased all-cause mortality vs. Alive; ^c^p-value: Deceased primary non-function of the liver vs. Group Alive.

MELD, Model for End: Stage Liver Disease; IAP, Intra-Abdominal Pressure; SOFA, Sequential Organ Failure Assessment; FB, Fluid Balance; L, Liter. Numbers are presented as n (%) or median and 25%; 75% IQR.
